# Recent advances in macrocyclic arenes-based fluorescent indicator displacement assays

**DOI:** 10.3389/fchem.2022.973313

**Published:** 2022-07-18

**Authors:** Qunpeng Duan, Fei Wang, Kui Lu

**Affiliations:** ^1^ School of Chemical and Printing-Dyeing Engineering, Henan University of Engineering, Zhengzhou, China; ^2^ School of Chemical Engineering and Food Science, Zhengzhou Institute of Technology, Zhengzhou, China

**Keywords:** calix[*n*]arenes, pillar[*n*]arenes, host-guest complex, fluorescence, indicator displacement assay

## Abstract

Macrocyclic arenes-based fluorescent indicator displacement assays (F-IDAs) offer a unique and innovative approach to chemosensing, taking molecular recognition in host-guest chemistry to a higher level. Because of their special architecture and versatile host–guest binding properties, macrocyclic arenes, principally calix[*n*]arenes and, in recent years, pillar[*n*]arenes, in combination with various fluorophores, are widely used in F-IDAs for the specific and selective sensing of cationic, anionic, and neutral analytes. In this paper, we review recent progress in the development of F-IDAs based on macrocyclic arenes and outline the prospects and remaining challenges relating to macrocyclic arenes-based F-IDAs.

## Introduction

Molecular sensors are considered a key component in the advancement of biological, environmental, and industrial sciences. Traditionally, the most widely used sensing assay has been the indicator spacer receptor approach (ISR), which involves the creation of covalent linkages between the indicator (fluorophore) and the receptor (Roberts, 1989). However, attaching the indicator to the receptor requires much synthetic work, and this is a major limitation of ISR. To avoid this problem, novel facile sensing assays through different non-covalent interactions have been developed (You et al., 2015; Mako et al., 2019; Hein et al., 2020), with indicator displacement assays (IDAs) being the most widely used, as well as offering a unique and innovative approach (Inouye et al., 1994; Nguyen et al., 2004). Displacement assays require that the indicator be able to reversibly bind to a receptor. The subsequent addition of a competitive analyte causes the displacement of the indicator from the receptor, regulating an optical signal if the indicator’s binding affinity to the receptor is smaller than that of the analyte.

**FIGURE 1:**
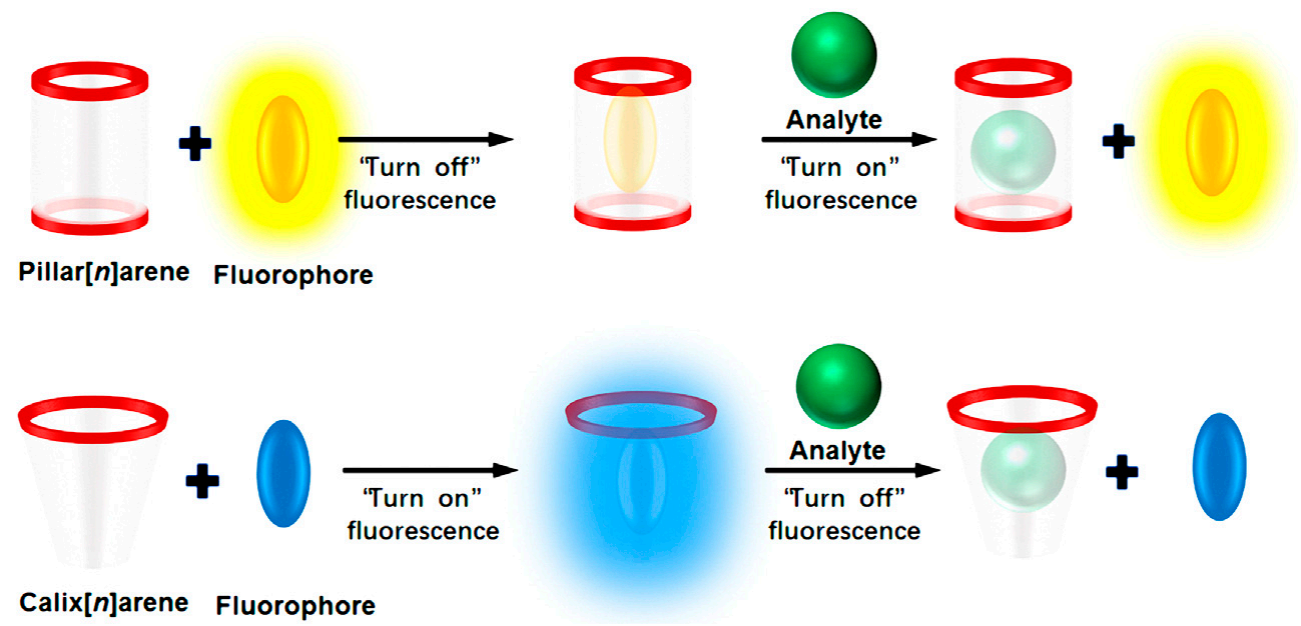
Cartoon depiction of an F-IDA which involves an integrated host-fluorophore sensor that disassembles in the presence of an analyte to produce a turn-on or turn-off fluorescence response.

Supramolecular chemistry, also known as host–guest chemistry, originated from the research of Pedersen, who was the first one to report the synthesis of crown ethers in 1967 (Pedersen, 1967). The field advanced rapidly, with an important development being the design of highly-selective macrocyclic hosts as receptors and their application in molecular sensing. The use of IDAs in supramolecular sensing was first proposed by Nguyen and Anslyn (2006), You and Anslyn (2012), You et al. (2015). Following these early efforts, the field has expanded to include colorimetric IDAs (C-IDAs) (Leontiev and Rudkevich, 2005), fluorescent IDAs (F-IDAs) (Martínez-Máñez and Sancenón, 2003), and metal complexing IDAs (M-IDAs) (Nguyen and Anslyn, 2006), etc. Of the different IDA approaches, F-IDAs provide the most convenient and efficient method to identify novel receptors able to bind to different target molecules (Bonizzoni, 2017). Calix[*n*]arenes, pillar[*n*]arenes, and their analogs—jointly referred to as “macrocyclic arenes”—are macrocyclic hosts formed by hydroxy-or alkoxy-substituted aromatic rings bridged by methylene or methenyl groups (Chen and Han, 2018). Macrocyclic arenes can provide the appropriate non-covalent interactions (such as hydrogen bonding, π-π or cation-π stacking, or hydrophobic or electrostatic interactions) to yield suitable complexation. F-IDAs have been created using synthetic macrocyclic arenes and a variety of fluorophores. The complexation of fluorophores by macrocyclic arenes causes readily detectable changes to their fluorescence properties, especially the intensity. The addition of an appropriate analyte can result in the displacement of the fluorophore from the complex to produce a detectable fluorescent response, converting the receptor-analyte binding event into an easily observable signal (Figure 1).

**FIGURE 2:**
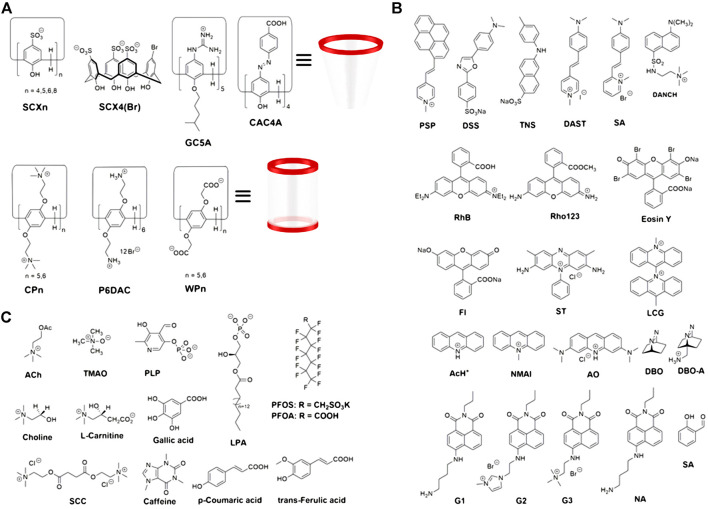
Chemical structures of **(A)** macrocyclic arenes as synthetic receptors, **(B)** fluorophores as indicators and **(C)** partial analytes detected as competitors.

This mini-review reports the progress of the development and application of macrocyclic arenes-based F-IDAs over the past 10 years, especially in the fields of analytical science and biological systems, focusing on two main types of macrocyclic arene, calix[n]arenes, and pillar[n]arenes. The structures of calix[*n*]arenes and pillar[*n*]arenes as synthetic receptors, fluorophores as indicators and analytes as competitors used in F-IDAs are shown in Figure 2. The objective of this review is to explore macrocyclic arenes-based F-IDAs in terms of their construction and detection mechanisms and major analytical applications.

## Calix[*n*]arenes-based fluorescent indicator displacement assays

Calix[*n*]arenes, first effectively synthesized and named by Gutsche (1998) in the late 1970s, were the third class of macrocycles to be developed. The calix[*n*]arenes (*n* ≥ 4) especially, which are comprised of hydroxy-substituted aromatic rings bridged with methylene groups at the *o*-positions of phenolic hydroxy groups, possess tunable scaffolds and controllable conformations for the binding of cationic and neutral organic guests (Boehmer, 1995). Due to their ability to alter the photophysical properties of fluorophores (Dsouza et al., 2011), calix[*n*]arenes have been used to form a host–guest complex for the determination of analyte concentrations *via* F-IDA (Ghale and Nau, 2014).

In 1996, Koh et al. (1996) proposed an artificial-signaling acetylcholine (**ACh**) receptor system that could easily illuminate the binding process of the cationic fluorophore to the *p*-sulfonatocalix[6]arene (**SCX6**) through a fluorescence intensity change. It was found that the fluorescence of the pyrene-modified pyridinium cation (**PSP**) is quenched upon binding to **SCX6** but then returns following the displacement of **SCX6** by **ACh**. In 2002, Zhang et al. (2002) also reported an F-IDA for determining **ACh** in a neutral aqueous medium based on **SCX8** and Rhodamine B (**RhB**). The formation of the **RhB**⊂**SCX8** complex brought about the fluorescence quenching of **RhB**, and the subsequent displacement of **RhB** by **ACh** caused an obvious increase in fluorescence emission. Later, Jin (2003) also developed an F-IDA to detect **ACh** in water based on **SCX8** and dansylcholine (**DANCh**). The fluorescence intensity of **DANCh** in an aqueous solution was increased by the complexation with **SCX8**, and the subsequent replacement of **DANCh** in the **DANCh**⊂**SCX8** complex by **ACh** greatly lowered the fluorescence intensity. This research provided a novel fluorometric approach to determine **ACh** (>10–4 M) with ATP and amino acids in a physiological salt solution.

In 2005, Bakirci et al. (2005) proposed a novel approach to detect inorganic cation binding *via* F-IDA based on the complexing of the receptor **SCX4** with the fluorescent indicator 2,3-diazabicyclo [2.2.2]oct-2-ene (**DBO**). Their research showed that fluorescence regeneration occurred in different metal ions, and binding between monovalent cations (alkali and ammonium) and **SCX4** was observed for the first time and quantitatively measured. Later in 2006, the same group also used the **DBO** and **SCX4** combination to detect choline and carnitine derivatives through F-IDA (Bakirci and Nau, 2006). They observed that adding **SCX4** to **DBO** solutions causes efficient fluorescence quenching, but adding choline and carnitine derivatives released **DBO** from the cavity of **SCX4**, leading to regeneration of its fluorescence. In 2007, **SCX4** and aminomethyl-substituted **DBO** (**DBO**-**A**) were also used for monitoring cationic products in amino acid decarboxylase-catalyzed reactions through F-IDA (Hennig et al., 2007). Upon binding to **SCX4**, the fluorescence intensity of **DBO**-**A** is quenched, and the signal is turned on after displacement of **DBO**-**A** from the **DBO**-**A**⊂**SCX4** complex by ammonium products. **SCX4** shows higher affinities to cationic alkylammonium products than amino acids, so adding amino acids would not affect the F-IDA. The authors demonstrated that this system is an efficient approach to the evaluation of the inhibitor for enzymatic transformation.

In 2011, Guo et al. (2011) reported a system utilizing **SCX4** and **SCX5** for the determination of **ACh** through F-IDA applicable to the real-time monitoring of acetylcholinesterase. Their research shows that lower concentrations of **ACh** binds with the macrocycle, leading to the release of cationic fluorophore lucigenin (**LCG**) and a fluorescence turn-on response. Although the bound **LCG** exhibited weak fluorescence, its displacement by **ACh** significantly increased emission, and, therefore, the conversion of acetylcholine to choline significantly decreased the fluorescence intensity. Later in 2016, **SCX4** and protonated cationic fluorophore acridine (**AcH** ^**+**^) were also used as the reporter pair for the detection of **ACh** through F-IDA. It was found that the binding of **AcH** ^**+**^ to **SCX4** causes a dramatic fluorescence “turn OFF,” which can be switched to a strong fluorescence “turn ON” through **ACh** addition (Sayed et al., 2016).

In 2012, Minaker et al. (2012) reported a mix-and-match F-IDA toolkit for use in responding to different histone code analytes, such as methylated lysine. This F-IDA system comprised the cationic fluorophores **LCG** and N-alkyl-pyridinium (**PSP**) modified by pyrene, as well as various calixarene hosts [**SCX4**, **SCX6**, and **SCX4**(**Br**)]. With the addition of cationic peptides, cationic fluorophores were emitted, causing a fluorescence turn-on response. The authors used these ensembles to identify the unmethylated, mono-, di-, and trimethylated lysine of a single histone tail sequence. On the other hand, Norouzy et al. (2015) also used **SCX4**-based F-IDA in living cells to absorb various bioorganic analytes, including protamine, choline, and **ACh**. Their research showed that tight binding of the fluorescent indicator **LCG** to the receptor **SCX4** caused fluorescence quenching of the **LCG**. The subsequent addition of organic analytes, such as choline, protamine, or **ACh**, could displace the indicator **LCG** from the receptor upon entry into living cells because of its high affinity with the receptor **SCX4**, thus, causing a fluorescence turn-on response. This research confirmed that F-IDA could be combined with other synthetic receptors to detect the uptake of bioorganic analytes in living cells.

In 2018, Gao et al. (2018) realized the discrimination of highly similar Glycosaminoglycans (**GAGs**) via F-IDA (Zheng et al., 2018a). An F-IDA comprising four reporter pairs and the cationic fluorophore eosin Y was developed. In the sensing array, the guanidinium and quaternary ammonium derivatives of calixarene act as the receptors. The complexation of calixarenes quenches the fluorescence of eosin Y, and, thereby, the competitive complexation of the six **GAG** analytes leads to fluorescence regeneration through the displacement of eosin Y from the calixarene receptors, achieving turn-on sensing. By implementing F-IDA on four reporter pairs for selective and sensitive detection of six **GAG** analytes, the authors derived a unique fluorescence response pattern for each **GAG**. This calixarene-based F-IDA strategy easily targets analyte libraries to achieve different sensing patterns for each analyte. In the same year, Guo and co-workers also applied F-IDA to the ultrasensitive selective detection of a cancer biomarker, lysophosphatidic acid (**LPA**), at a nanomolar level by a turn-on fluorescence response with guanidinium-modified calix[5]arene (**GC5A**) as the receptor and fluorescein (**FI**) as the fluorescent indicator (Zheng et al., 2018b). They observed that **FI** fluorescence emission quenching occurred after the binding of **FI** with **GC5A**, and subsequent displacement of **FI** from **GC5A** through competitive binding by **LPA**, causing a turn-on fluorescence response. In another study, the same group also the **GC5A**-**FI** pair in the sensitive, selective, and label-free detection of bisphosphonates (**BPs**) in buffer solution and artificial urine via F-IDA, with the replacement of **FI** in the complex by **BPs** triggering a turn-on fluorescence response (Gao et al., 2018). This label-free sensing strategy shows application potential for the real-time monitoring of **BPs** concentration in urine and pharmacokinetic research. Later in 2019, the same group utilized the **GC5A**-**FI** pair for the sensitive and quantitative detection of **PFOA** and **PFOS** in contaminated water *via* F-IDA (Zheng et al., 2019). They also reported a sensing strategy suited to the turn-on fluorescence detection for metabolite trimethylamine N-oxide (**TMAO**) through F-IDA using **GC5A** and **FI**, demonstrating an inexpensive, convenient, label-free, and sensitive approach to detecting **TMAO**, which provides a new method for **TMAO** detection in clinical studies (Yu et al., 2019). The **GC5A** and **FI** reporter pair was also chosen to detect turn-on fluorescence in enzymatic substrate Pyridoxal-5′-phosphate (**PLP**) through F-IDA. (Yue et al., 2019).

Guo and co-workers proposed a simple F-IDA strategy to detect hypoxia in living cells based on an azocalix[4]arene (**CAC4A**) host and the fluorescent guest rhodamine 123 (**Rho123**) (Geng et al., 2019). Bioreductive enzymes capable of reducing various organic functionalities can be over-expressed in a hypoxic environment. **Rho123** exhibited fluorescence quenching upon being located inside the cavity of **CAC4A**, and in a hypoxic environment, the azo groups of **Rho123**⊂**CAC4A** are reduced and transformed to amino groups, causing the release of **Rho123** and a fluorescence turn-on response (Geng et al., 2019).

## Pillar[*n*]arenes-based fluorescent indicator displacement assays

Pillar[*n*]arenes, also known as “pillararenes,” are new macrocyclic hosts, being studied for the first time by Ogoshi et al. (2008). These macrocyclic hosts are usually produced by the condensation of hydroquinone with paraformaldehyde (Ogoshi et al., 2016). They are widely studied because of their synthetic accessibility and pillar-shaped three-dimensional structures. Their symmetrical rigid pillar-shaped cavity, electron-rich cavity, easy modification, and special host-guest recognition characteristics have led researchers to favor them over traditional calix[n]arenes for building F-IDAs to detect different analytes.

In 2014, Wang et al. (2014) reported the first pillararene-based F-IDA, the water-soluble carboxylato-pillar[5]arene (**WP5**), as a macrocyclic host for the detection of paraquat via F-IDA. They used *N*-methylacridinium iodide (**NMAI**), a fluorophore, as the fluorescent indicator and found that, at pH 7.4, paraquat displaced the fluorophore from the **WP5** cavity, resulting in a prominent emission increase. Later in 2015, Bojtár et al. (2015) reported a fluorescence spectroscopic study on the host-guest interaction of three different pyridinium-based stilbazolium dyes and **WP5** in an aqueous solution. The resulting **WP5** and 4-dimethylaminostyryl-N-methylpyridinium iodide (**DAST**) complex was successfully applied as an F-IDA-based probe for the sensitive detection of paraquat. In another study, Bojtár et al. (2016) presented a supramolecular sensory system consisting of **WP5** and a 4-amino-1,8-naphthalimide cationic fluorophore having three anchors (**G1**, **G2**, and **G3**) that they used to selectively investigate basic amino acids via F-IDA. Later in 2019, based on their previous studies, Bojtár et al. (2016) also evaluated the complexation of three fluorescent indicators-a pyridinium-based stilbazolium dye (**DAST**) and two 4-amino-1,8-naphthalimide fluorophores with positively charged anchors (**G2** and **G3**)-with carboxylato-pillar[6]arene, **WP6**. The stilbazolium dye gave turn-on, and the two naphthalimide derivatives turn-off fluorescence responses upon complexation. The two **WP6**-indicator supramolecular systems were used in the F-IDAs selective detection of histamine (Paudics et al., 2019). In another example, Hua et al. (2018) discovered a new **WP6**-based pillararene-indicator complex utilizing an aromatic fluorophore acridine orange (**AO**). Under the system, choline bound strongly to the **WP6** cavity, displacing the already captured **AO**, causing emission enhancement. The constructed pillararene-indicator system was used to detect choline compounds and monitor enzymatic reactions. In the meantime, Pan et al. (2018) proposed a simple and rapid F-IDA method to detect Succinylcholine Chloride (**SCC**) based on naphthalimide dye (**NA**) or stilbazolium dye (**SA**) and the **WP5** complex. In this F-IDA study, upon addition of **SCC** to the **NA**⊂**WP5** or **SA**⊂**WP5** solution, there was a recovery of the fluorescence intensity, bringing about the recognition of **SCC** due to competitive supramolecular displacement between **SCC**, **NA**, and **SA** for **WP5**. Recently, the authors of this mini-review proposed the use of F-IDA to perform selective detection of caffeine based on WP6 and the fluorophore safranine T (**ST**). The addition of caffeine to the **ST**⊂**WP6** complex brings about **ST** displacement because of the higher binding affinity, with a turn-off fluorescence response (Duan et al., 2021).

Cationic water-soluble pillar[n]arenes containing an ammonium group have also been used to construct F-IDA to detect various analytes. In 2016, Hua et al. (2016) described a turn-on fluorescence switch based on the host-guest complexation between a water-soluble ammonium pillar[5]arene (**CP5**) and a salicylaldehyde (**SA**). **CP5** encapsulated **SA** to promote deprotonation of **SA** and generate a highly fluorescent host-guest complex. **SA** deprotonation and deaggregation induced by complexation led to a significant fluorescence increase in the **SA** solution. This turn-on fluorescence switch was also used to detect F-IDA in phenols and chlorophenols. Later in 2017, Bojtár et al. (2017) employed the water-soluble ammonium pillar[6]arene (**CP6**) receptor with a larger cavity and a fluorescent indicator dapoxyl sodium sulfonate (**DSS**) for F-IDA. The complexation between **CP6** and **DSS** produced a significant increase in fluorescence intensity. This system was selective for ATP detection over ADP/AMP/GTP through the ATP-induced displacement of the **DSS** on the **CP6**. This research was the first to demonstrate the importance of cationic pillararene-based F-IDA in detecting analytes with biological importance. Recently, the authors of this mini-review reported a novel fluorescence activation switch based on the host-guest complexation between a fluorescent indicator probe 6-*p*-toluidinylnaphthalene-2-sulfonate (**TNS**) and water-soluble pillar[6]arene dodecyl-ammonium chloride (**P6DAC**). The complexation remarkably increased the fluorescence intensity, and adding food additives (p-coumaric acid, trans-ferulic acid and gallic acid) to the **TNS**⊂**P6DAC** complex results in the displacement of **TNS** and thus causes a turn-off fluorescence response, which was employed for the F-IDA detection of phenolic food additives (Duan et al., 2022).

## Conclusion and outlook

In this mini-review, we have attempted to highlight the recent advances and future prospects of F-IDAs using macrocyclic arenes as molecular recognition units. Direct sensing based on F-IDAs was explored for various analytes, such as cations, anions, small neutral molecules, or bioanalytes, using calix[*n*]arenes and pillar[*n*]arenes. Though great progress has been achieved in the development of macrocyclic arenes-based F-IDAs, there are still relatively few F-IDAs that can be truly applied to real-world environments. However, progress to date suggests that, through continued effort, more macrocyclic arenes and fluorescent indicators with better performance will be synthesized, and, in the future, F-IDAs based on macrocyclic arenes will become more reliable and versatile tools that address an ever-widening scope of real-world problems, such as the detection of diseases and providing environmental monitoring.
